# Engineering production of functional scFv antibody in *E. coli* by co-expressing the molecule chaperone Skp

**DOI:** 10.3389/fcimb.2013.00072

**Published:** 2013-11-06

**Authors:** Rongzhi Wang, Shuangshuang Xiang, Youjun Feng, Swaminath Srinivas, Yonghui Zhang, Mingshen Lin, Shihua Wang

**Affiliations:** ^1^The Ministry of Education Key Laboratory of Biopesticide and Chemical Biology, College of Life Sciences, Fujian Agriculture and Forestry UniversityFuzhou, China; ^2^Department of Microbiology, University of Illinois at Urbana-ChampaignChampaign, IL, USA; ^3^Department of Biochemistry, University of Illinois at Urbana-ChampaignChampaign, IL, USA; ^4^TA Instruments-Waters LLCShanghai, China

**Keywords:** *Vibrio parahaemolyticus*, scFv, co-expression, solubility, production

## Abstract

Single-chain variable fragment (scFv) is a class of engineered antibodies generated by the fusion of the heavy (V_H_) and light chains (V_L_) of immunoglobulins through a short polypeptide linker. ScFv play a critical role in therapy and diagnosis of human diseases, and may in fact also be developed into a potential diagnostic and/or therapeutic agent. However, the fact that current scFv antibodies have poor stability, low solubility, and affinity, seriously limits their diagnostic and clinical implication. Here we have developed four different expression vectors, and evaluated their abilities to express a soluble scFv protein. The solubility and binding activity of the purified proteins were determined using both SDS-PAGE and ELISA. Amongst the four purified proteins, the Skp co-expressed scFv showed the highest solubility, and the binding activity to antigen TLH was 3-4 fold higher than the other three purified scFv. In fact, this scFv is specific for TLH and does not cross-react with other TLH-associated proteins and could be used to detect TLH directly in real samples. These results suggest that the pACYC-Duet-*skp* co-expression vector might be a useful tool for the production of soluble and functional scFv antibody.

## Introduction

Single-chain variable fragment (scFv) is a fusion protein formed by engineering the association of the V_H_ and V_L_ domains of the antibodies with a short polypeptide linker. scFv specific to any particular antigen may be easily generated by Phage display (Wang et al., 2006), and this scFv have been developed to select molecular targets in cancer research such as in lymphatic invasion vessels, colon cancer and hepatocarcinoma (Rinderknecht et al., 2010; Sakai et al., 2010; Sommaruga et al., 2011). Furthermore, scFv has been extensively used to generate ligands for detecting pathogenic germs *in vitro* or *in vivo* (Wang et al., 2007, 2008a, b; Zhang et al., 2010; Cattepoel et al., 2011). In comparison to polyclonal antibodies or the hybridoma technology, scFv antibody may be easily manipulated for improving specificity and affinity, thereby reducing the production cost (Coia et al., 2001; Krag et al., 2006). Combing scFv with selection panning strategies, we were able to character the binding properties of scFv and investigate the potential use of these scFv as diagnostic tools or therapeutic agents (Eisenhardt et al., 2007; Rothe et al., 2007). However, these above mentioned applications of scFv were limited by drawbacks such the formation of inclusion bodies, which often lead to low binding activity, unstable structure and are cytotoxic to host cells.

Currently, the soluble expression of scFv antibody remains an awkward plight, so the majority of the work in this field focuses on developing a strategy based on molecular manipulation to improve the stability and solubility of scFv antibody. Till today, a number of methods have been used to express the scFv antibody, including expression of affinity tag fusion (Esposito and Chatterjee, 2006), co-expression of molecular chaperones, and folding modulators (De Marco and De Marco, 2004; Sonoda et al., 2011), extracellular accumulation in a defined medium (Fu, 2010), refolding scFv using detergent and additive (Kudou et al., 2011) and expression in different host systems (Goulding and Perry, 2003). Amongst of these methods, expression of affinity tags fusion protein is the common method to improve the solubility of target proteins. Previously, some affinity tags such as thioredoxin (TRX) (Nygren et al., 1994), maltose binding protein (MBP) (Nallamsetty and Waugh, 2006), N-utilization substance A (NusA) (Fox and Waugh, 2003), bacteriophage T7 protein kinase gene (T7PK) (Jurado et al., 2006), small peptide tags (SET) (Davis et al., 1999), monomeric mutant of the Ocr protein of bacteriophage T7 (Mocr) and glutathione S-transferase (GST) were used to enhance the solubility of some of the partner proteins to which they were attached (DelProposto et al., 2009). Unfortunately, the tags needed to be cleaved as the large tags usually interfered with the folding of their partner protein and made them more difficult to assay for activity and for functional research (Esposito and Chatterjee, 2006). Besides, the partner proteins often remained insoluble when the fusion tags were removed, and the entire process of tags removal is costly and laborious (Esposito and Chatterjee, 2006). Though the use of detergents and additives to refold the target protein can assist in making protein soluble, there is still no guarantee that these methods will be suitable for every protein of interest. When it comes to expression system, though a number of them, such as *E. coli*, Yeast, mammalian, insect cell, wheat germ cell-free expression system, and plant-based expression system (Goulding and Perry, 2003; Greene, 2004; Daly and Hearn, 2005; Guild et al., 2011), are available for protein production, the *E. coli* host system is widely regarded as the most suitable host for the expression of recombinant antibody fragments (Wang et al., 2008a, b). Compared to other host systems, the *E. coli* system is an economical, shows faster growth and is easier to manipulate genetically (Sushma et al., 2011).

It was also reported that the solubility and affinity of scFv was improved by co-expression of molecular chaperones such as Skp, Dsbc, and FkpA (Ow et al., 2010; Sonoda et al., 2011). In some cases, co-expression of molecular chaperone not only improves the soluble expression but also increases the cell viability (Ow et al., 2010). Skp is a key periplasmic chaperone (18 kDa) that plays an important role in folding and assembling of outer membrane proteins in *E. coli*, and reports have shown that the co-expression of Skp facilitates correct folding of the expressed protein and enhances the solubility and affinity of proteins (Hayhurst and Harris, 1999; Hayhurst et al., 2003; Sonoda et al., 2011).

In this study, we investigate the effects of the molecular chaperone Skp on the level of expression, solubility and antigen-binding activity of scFv. The co-expression vector pACYC-Duet containing two multiple cloning sites (MCS), was used for the construction and expression of two target genes in an appropriate host strain. By co-expression of a periplasmic chaperone (Skp) with scFv, a relatively high amount of soluble scFv antibody was successfully obtained.

## Materials and methods

### Materials

*V. parahaemolyticusCGMCC1.2164, V. parahaemolyticusCGMCC1.1614, V. parahaemolyticusCGMCC1.1615, V. parahaemolyticus*CGMCC1.1616 were purchased from the Institute of Microbiology, Chinese Academy of Sciences (Beijing, China). All other strains were from Fujian Agriculture and Forestry University (Fujian, China). The plasmids pET28a(+) and pET32a(+) were purchased from Novagen (Madison, WI, USA), and the vector pACYC-Duet-1 was generously provided by Professor Lijun Bi (Institute of Biophysics, Chinese Academy of Sciences, Beijing China). The vector pGEPi was generously provided by Dr Mengfei Ho (University of Illinois at Urbana-Champaign, USA). DNA restriction enzymes were purchased from Promega (USA). Taq DNA polymerase and T4 DNA ligase were purchased from Takara (Dalian, China). Anti 6×His tag monoclonal antibody was purchased from Abgent (USA), and horseradish peroxidase (HRP)-labeled goat anti-mouse IgG was from Boster Biological Technology Co. (Wuhan, China). All oligonucleotides were listed in Table 1, and all other reagents used were of analytical reagent grade.

**Table 1:** **Primers used in the present study**.

**Primers**	**DNA sequence**
Skp-F	GGCGAGATCTGACGAAAAAGTGGTTATTAGCTG
Skp-R	CGGCTCGAGTTATTTAACCTGTTTCAGTACGTC
scFv-1-F	AAGAATTCATGGCCCAGGTCAAACTGCAGGAG
scFv-1-R	CCCGCAAGCTTCCGTTTTATTTCCAGCT
scFv-2-F	AAGAATTCAGCCCAGGTCAAACTGCAGGAG
scFv-2-R	CCCGCAAGCTTCCGTTTTATTTCCAGCT
TLH-F	AAACTGGTACCGGTAGAAATGATGAAAAAAACAATCAC ACTATTAACTGCATTAC
TLH-R	GAACCTGCGGCCGCACCAGAACCGAAACGGTACTCGG CTAAGTTGTTGCTAC

### Expression and purification of TLH antigen

The *V. parahaemolyticus*(CGMCC1.2164) genome was used as a template to amplify the *tlh* gene[GeneBank:GU971665.1] (Wang et al., 2011). Primers with *Kpn* I and *Not* I restriction enzymatic sites were designed for cloning the *tlh* gene into pGEPi vector. The constructed pGEPi-*tlh* vector was transformed into *E. coli* BL21 by electroporation, and a single colony from the selection plate was inoculated into 5 mL LB liquid media containing 100 μg/mL ampicillin for the expression of TLH antigen. The expressed protein contains HA and Myc tags at the C-terminal (without 6×His tag). Expression of the target protein was induced by adding 1 mM IPTG when the culture reached an OD_600_ of 0.8, and the induced cells were grown at 37°C for overnight, and then harvested by centrifugation. After SDS-PAGE, the expressed proteins were stained using 0.3 M CuCl_2_ by shaking for 5 min at 37°C, and visualized against a black background. The target TLH protein bands were cut and mashed, and mixed with 1 mL PBS. The mixture solution was dialyzed with Gly/Tris buffer and the supernatant was harvested by centrifugation, and the extracted supernatant containing the TLH protein was visualized by SDS-PAGE. Protein concentration was determined by the BCA protein assay kit.

### Construction of expression vectors for scFv

pET28a(+), pET32a(+), and pACYC-Duet vectors were used for the construction of expression vectors with the scFv proteins fused to 6×His tag, 6×His-TRX, and 6×His tag, respectively. For co-expression, a recombinant vector pACYC-Duet-*skp* was first constructed. The DNA *skp* gene[GeneBank:U00096.2] encoding the molecular chaperone was amplified using the primers Skp-F and Skp-R, and JM109 genomic DNA was used as template. The amplified DNA fragment was digested by *Bgl* II and *Xho* I, and ligated the pACYC-Duet vector digested with the same restriction enzymes. To construct four different formats of expression vectors, the segment encoding anti-TLH scFv was amplified using the template vector pCANTAB-5E-*scFv* (Wang et al., 2012) and the following primers (scFv-1-F and scFv-1-R for scFv-1; scFv-2-F and scFv-2-R for scFv-2). The amplified scFv-1 fragment was digested with *EcoR* I and *Hind* III, and ligated with pET28a(+) and pET32a(+) vectors digested with *EcoR* I and *Hind* III, and the resultant vectors were designated as pET28a-*scFv* and pET32a-*scFv*, respectively. The amplified scFv-2 fragment was digested with *EcoR* I and *Hind* III, and ligated with pACYC-Duet and pACYC-Duet-*skp* vectors digested with the *EcoR* I and *Hind* III, and the resultant vectors were designated as pACYC-Duet-*scFv* and pACYC-Duet-*scFv*-*skp*, respectively.

### Expression and soluble analysis of scFv antibodies

For protein expression, the recombinant plasmids were transformed into *E. coli* BL21 by electroporation, and a single colony from the selection plate was inoculated into 5 mL LB liquid media containing either 100 μ g/mL ampicillin or 50 μ g/mL kanamycin or 34 μ g/mL chloramphenicol. The culture was incubated overnight with shaking at 37°C, and then transferred to a larger-scale LB media (1 mL culture transferred into 500 mL fresh LB). Expression of the target protein was induced by adding 1 mM IPTG when the culture reached an OD_600_ of 0.8. Cells were grown for an additional 12 or 32 h (only for pACYC-Duet-*scFv*-*skp*/BL21) at 16°C, and then harvested by centrifugation. To ensure the accuracy of the experiment, four different derived cultures were adjusted to the same concentration for samples treatment, and the treated proteins were analyzed by SDS-PAGE using 12% (v/v) polyacrylamide gels.

### Purification and identification of anti-TLH scFv antibodies

The purification of the expressed anti-TLH scFv was performed using Ni^2+^ affinity chromatography. The expressed product was first harvested by centrifugation (10000 r/min) at 4°C for 10 min, followed by addition of 5 mL of the binding buffer (50 mM NaH_2_PO_4_, 300 mM NaCl, 1 mM imidazole, 0.05% Tween 20) after the removal of the supernatant. The complex was then mixed gently, sonicated and then centrifuged at 4°C for 10 min. The supernatant was collected and loaded onto a 3 mL Ni^2+^-NTA column, which was equilibrated with the binding buffer before the purification. After allowing the sample to flow-through, the column was washed twice with the wash buffer (100 mL/time), and the wash fraction was collected for SDS-PAGE analysis. After the wash, the retained protein of interest was eluted with 0.5 mL elution buffer 4 times, and the eluate was collected and analyzed by SDS-PAGE and verified by Western blotting. Protein concentration was determined by the BCA protein assay kit.

### Elisa analyses

The activity of the purified scFv products was determined by ELISA. The dialyzed TLH antigen (without 6×His tag) was used to coat the 96 well plates at 4°C for overnight. After blocking and washing the ELISA plate, the purified scFv products derived from four different formats of plasmids were added to the reaction wells and incubated at 37°C for 2 h. Then, the anti-His tag antibody was added to the reaction wells and incubated at 37°C for 2 h. The binding activity of the purified scFv was detected by using a HRP conjugated anti-mouse IgG antibody. The enzyme reaction was then performed with TMB as a substrate and color development was terminated with 2 M H_2_SO_4_. Absorbance at 450 nm was measured using a microplate reader.

Similarly, ELISA was also used for the detection of bacterial TLH. Totally, eleven different types of *V. parahaemolyticus* strains, seven *Vibrio* strains from other species, and four non-*Vibrio* bacterial strains were tested using ELISA. The bacterial cultures were grown in 5 mL APW medium without shaking at 37°C overnight, and then their supernatants were collected by centrifugation (8000 r/min, 15 min). The supernatants were added to a 96 well plate (100 μL/well) to perform ELISA as described before.

### Western blotting

To further confirm the binding activity of scFv (co-expression), western blotting was performed as described by Singh et al. (2010) with minor modifications. Briefly, the extracted TLH was transferred from a SDS-PAGE gel onto a polyvinylidene difluoride (PVDF) membrane, and the membrane was treated with purified scFv antibody and anti-His tag antibody. After washing and blocking, the membrane was subsequently incubated with HRP-conjugated anti-mouse IgG antibody. Signals were visualized by enhanced chemiluminescence (ECL).

### Analyses of anti-TLH scFv specificity

ELISA was performed to determine the specificity of the expressed anti-TLH scFv. BSA, KLH, and associated *V. parahaemolyticus* antigens (Vp1668 and YscF) were coated on 96-well plates (5 μg/mL, 100 μL/well) at 4°C for overnight, respectively. After the blocking and washing, the purified scFv (pACYC-Duet-*scFv*-*skp*) was added to the reaction wells and incubated at 37°C for 2 h. Then the anti-His tag antibody was added to the reaction wells and incubated at 37°C for 2 h. The specificity of the scFv was detected with the HRP conjugated anti-mouse IgG antibody.

### Affinity determination of skp co-expression scFv by microcalorimetry

The bio-molecular interaction and affinity of the scFv to TLH was measured using a TA-instrument Nano ITC-LV. The measurement was automatically monitored by data acquisition software supplied along with the instrument. All the measurements were carried out at 25°C. The affinity constant was calculated using Origin software supplied with the Nano ITC-LV.

## Results

### Construction of fusion expression vectors carrying scFv

**Figure 1:**
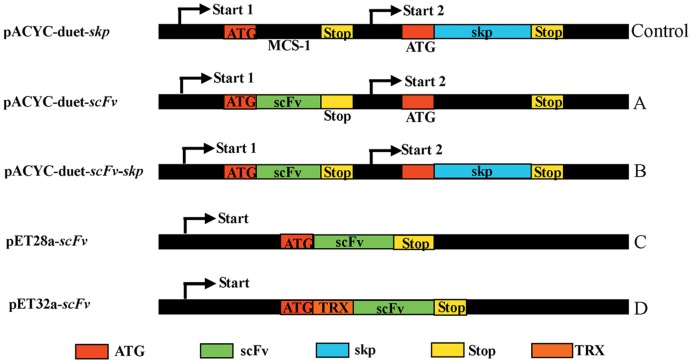
**Constructs of scFv with different fusion formats used in this study.** MCS, Multiple cloning sites; Stop, stop codon; scFv, single chain variable fragment; Skp, molecular chaperone Skp; TRX, thioredoxin.

In this study, four different expression vectors pACYC-Duet-*scFv*, pACYC-Duet-*scFv*-*skp*, pET28a-*scFv*, and pET32a-*scFv* were constructed (Figure 1), and transformed into *E. coli* BL21 to investigate their effects on solubility and activity of the expressed scFv. Among of four expression vectors, pET28a is the basic vector for protein expression, pET32a contains a TRX tags that can enhance the solubility of protein. While pACYC-Duet-*skp* contains two MCS sites for different DNA insertions, and all the expression vectors contains a 6×His tag for protein purification and ELISA detection. To analyze the function of Skp on soluble expression, the vector pACYC-Duet-*skp* was first constructed by introducing the *skp* gene into the multiple cloning sites 2 with restriction enzyme sites *Bgl* II and *Xho* I. Then the scFv gene was cloned into the multiple cloning sites 1 via *EcoR* I and *Hind* III, and the resulting pACYC-Duet-*scFv-skp* was used for the expression of both proteins (Skp and scFv) in *E. coli* BL21, with the expressed scFv contains a 6×His tag at C-terminal. The expression vector pET28a-*scFv* encodes scFv with two 6×His tags, one each at the N- and C-terminal, respectively, while the expression vector pET32a-*scFv* encodes a TRX-scFv fusion protein with two 6×His tags, one each at the N-terminal and C-terminal, respectively.

### Expression and purification of TLH antigen and scFV

**Figure 2:**
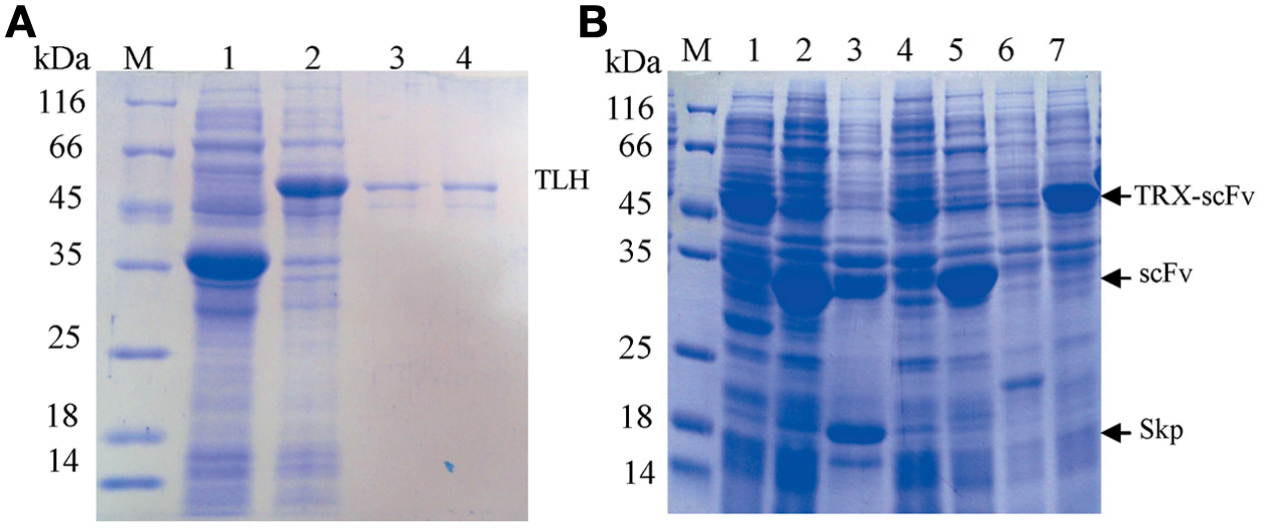
**SDS-PAGE analysis. (A)** SDS-PAGE analysis of expressed TLH antigen protein. Lane 1: Control (empty vector pGEPi, IPTG induced); Lane 2: pGEPi-*tlh* expressed product (IPTG induced); Lane 3, 4: Extracted TLH protein (without 6×His tag); **(B)** Expression of different scFv constructs in *E. coli* BL21. M: Molecular weight of marker proteins; Lanes 1, 4, and 6: negative control (pACYC-Duet, pET28a, and pET32a, respectively); Lane 2, 3, 5, and 7: the expressed products of pACYC-Duet-*scFv*, pACYC-Duet-*scFv-skp*, pET28a-*scFv*, and pET32a-*scFv*, respectively.

After the TLH protein was expressed, the total protein was analyzed by SDS-PAGE, and the gel was stained using 0.3 M CuCl_2_, and visualized against a black background. The target TLH protein bands were cut and mashed with PBS buffer. After harvested by centrifugation, the extracted supernatant containing the TLH protein was visualized by SDS-PAGE. As shown in Figure 2A, the TLH antigen protein was expressed successfully (Figure 2A, lane 2), and the target TLH protein was extracted with a high purity (Figure 2A, lane 3, 4). The expressed TLH protein contains HA and Myc tags (without 6×His tag), and was used as the immobilized antigen during ELISA.

Four constructed vectors were used to transform *E. coli* BL21 (DE3) by electroporation for the expression of their respective target proteins. The transformants were grown in test-tube cultures and induced with 0.5 mM IPTG at 16°C for 12 or 36 h, and the expressed products were analyzed by SDS-PAGE. As shown in Figure 2B, the target proteins were expressed successfully in every kind of vector, and that the apparent molecular weight of each target scFv was consistent with its corresponding theoretical molecular weight.

### Expression and analysis of solubility of scFv

**Figure 3:**
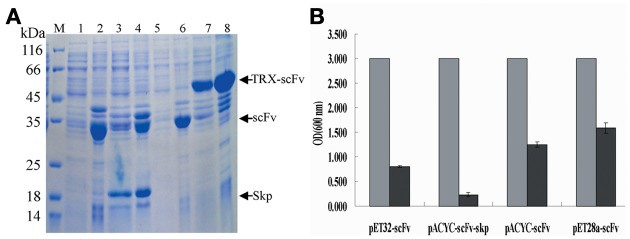
**Analysis of solubility of expressed products. (A)** Analysis of solubility of the expressed protein by SDS-PAGE. Lane 1, 3, 5, and 7: the soluble cell lysates for pACYC-Duet-*scFv*, pACYC-Duet-*scFv-skp*, pET28a-*scFv*, and pET32a-*scFv*, respectively; Lane 2, 4, 6, and 8: the insoluble cell lysates for pACYC-Duet-*scFv*, pACYC-Duet-*scFv-skp*, pET28a-*scFv*, and pET32a-scFv, respectively. **(B)** Cell lysates analysis by OD_600_ assay (gray bar indicates the concentration of cells after protein expression; black bar indicates the concentration of cell lysates after ultrasonication).

To test the solubility of expressed proteins, we lysed the cells by ultrasonication after protein expression, and the results were analyzed by SDS-PAGE and OD_600_ value. As shown in Figure 3A, Skp co-expression (Figure 3A, lane 3) and TRX fusion expression (Figure 3A, lane 7) were the best choices with respect to protein solubility, in comparison to the other two fusions (Figure 3A, lane 1, 5) where a large portion proteins were expressed in an insoluble forms (Figure 3A, lane 2, 6). This was further confirmed by the measure of OD_600_ (Figure 3B). These results demonstrated that the solubility of expressed protein was improved by either TRX fusion or Skp co-expression.

### Purification and identification of anti-TLH scFv

**Figure 4:**
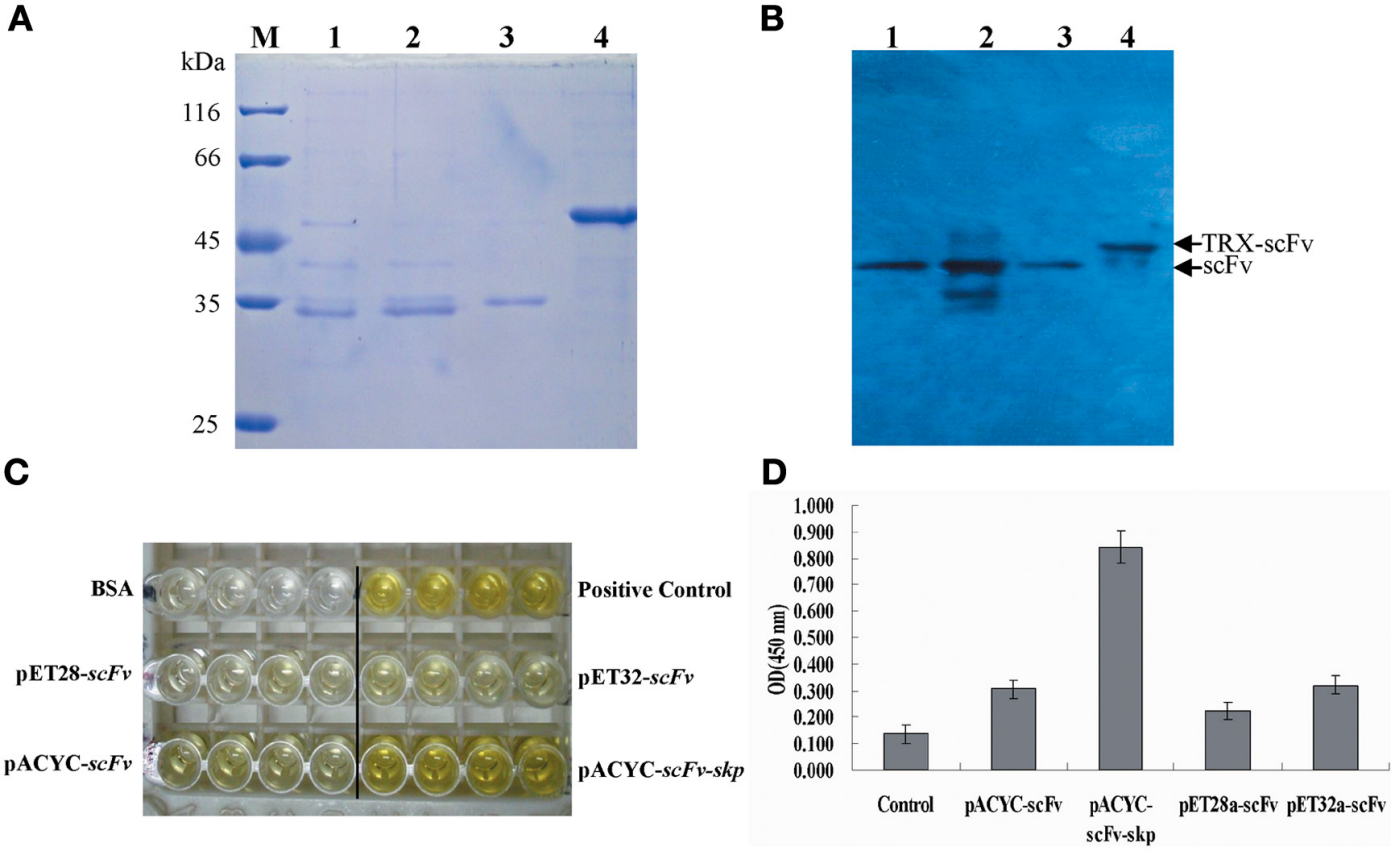
**Protein purification and identification. (A)** Protein purification. The purified protein was determined by SDS-PAGE. M: Molecular weight of marker proteins; Lane 1–4: the purified proteins for pACYC-Duet-*scFv*, pACYC-Duet-*scFv*-*skp*, pET28a-*scFv*, and pET32a-*scFv*, respectively. **(B)** Western blotting results. Lane 1–4: the detected bands for pACYC-Duet-*scFv*, pACYC-Duet-*scFv*-*skp*, pET28a-*scFv*, and pET32a-*scFv*, respectively. **(C)** ELISA. TLH antigen were coated on 96-well plates in triplicate (5 μ g/mL, 100 μ L/well), and the purified scFv proteins were added to the reaction wells after blocking and washing. The binding activities of the four purified proteins were determined using an anti-6×His tag antibody. **(D)** Quantitative result of ELISA^*^, *P* < 0.05, compared with control antibody treatment.

To purify scFv protein, cells were grown for an additional 12 or 32 h with IPTG induction at 16°C and then harvested by centrifugation. Protein purification was performed using Ni^2+^ affinity chromatography, and the purified proteins were visualized by SDS-PAGE using 12% (v/v) polyacrylamide gels. The concentrations of purified proteins from four different constructs are 0.13, 0.29, 0.115, and 0.38 mg/mL, respectively. After SDS-PAGE analysis, the target protein was further transferred onto a PVDF membrane, and anti-6×His tag antibody was used to confirm the presence of the expressed scFv protein. Protein purification and western blotting results showed that all four different fusion proteins were purified successfully (Figure 4A), and that all four could be recognized by the anti-6×His tag antibody during western blotting (Figure 4B).

To compare the effects of different fusion tags on the production of functional protein, the four purified proteins were assessed for their binding activity to the TLH antigen by ELISA. As seen in Figures 4C,D, amongst of four different proteins, the purified protein derived from the pACYC-Duet-*scFv*-*skp* vector showed the highest binding activity to TLH antigen, with the binding activity being 3–4 folds higher than the others. Although TRX fusion expressed scFv had higher solubility, the binding activity of TRX fusion protein was barely satisfactory. It was lower than that of scFv obtained by Skp co-expression, and only showed the similar binding activity similar to the scFv expressed from the plasmids pACYC-Duet-*scFv*. In fact, the scFv expressed from the pET28a-*scFv* showed a very low binding activity to antigen TLH.

### Specificity analysis and affinity determination of scFv obtained by Skp co-expression

**Figure 5:**
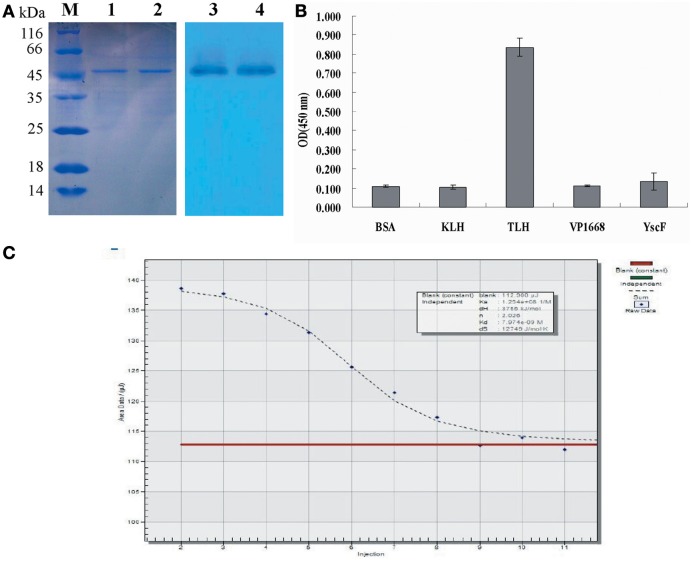
**Western blotting and affinity determination. (A)** Western blotting analysis of the binding activity of scFv obtained by Skp co-expression to the TLH antigen. Left panel: SDS-PAGE results for the extracted TLH; Lane M: Protein molecular weight markers; Lanes 1, 2: extracted TLH. Right panel: Western blotting results; Lanes 3, 4: TLH band at 47 kDa bound by scFv obtained by Skp co-expression. **(B)** Specificity analysis of scFv obtained by Skp co-expression. BSA, KLH, and associated antigen proteins (VP1668 and YscF) of *V. parahaemolyticus* were coated on 96-well plates in triplicate (5 μ g/mL, 100 μ L/well). The purified scFv were added to the reaction wells and incubated for 2 h at 37°C. Specificity of Skp co-expressed scFv was determined using an anti-6×His tag antibody. **(C)** Affinity Determination of Skp co-expressed scFv. The affinity of anti-TLH Skp co-expressed scFv for the TLH antigen was studied by Microcalorimetry using the Nano ITC-LV. The measured data was used for the quantitative determination of the affinity constant of the anti-TLH Skp co-expressed scFv antibody.

As shown in Figure 5A, a clear band was revealed via western blotting (Figure 5A, lane 3, 4), and the result demonstrated that the TLH band at 47 kDa could be recognized by scFv obtained by Skp co-expression. To further determine the specificity of Skp co-expressed scFv protein to TLH antigen, ELISA was carried out. As shown in Figure 5B, Skp co-expressed scFv was found to be specific to TLH, while there was no cross binding to any other antigen-associated proteins such as BSA, KLH, Vp1668, and YscF. These results indicated that the co-expressed scFv could recognize TLH specifically and be used as an antibody reagent to detect TLH.

To further probe the interaction of Skp co-expressed scFv and TLH antigen, the affinity of anti-TLH scFv for the TLH antigen was studied by Microcalorimetry using the Nano ITC-LV. The measured data was used for the quantitative determination of the affinity constant of the anti-TLH scFv antibody. It was observed that the TLH antigen could be recognized by Skp co-expressed scFv, and the binding activity was dose dependent. The calculated affinity constant of anti-TLH scFv was 1.254 × 10^8^ L/mol as determined by Origin software (Figure 5C). The above result was consistent with our earlier results obtained by ELISA, and the magnitude of the affinity constants consistently remain at the level of 10^8^ (Wang et al., 2012).

### Elisa detection of TLH in bacteria

**Figure 6:**
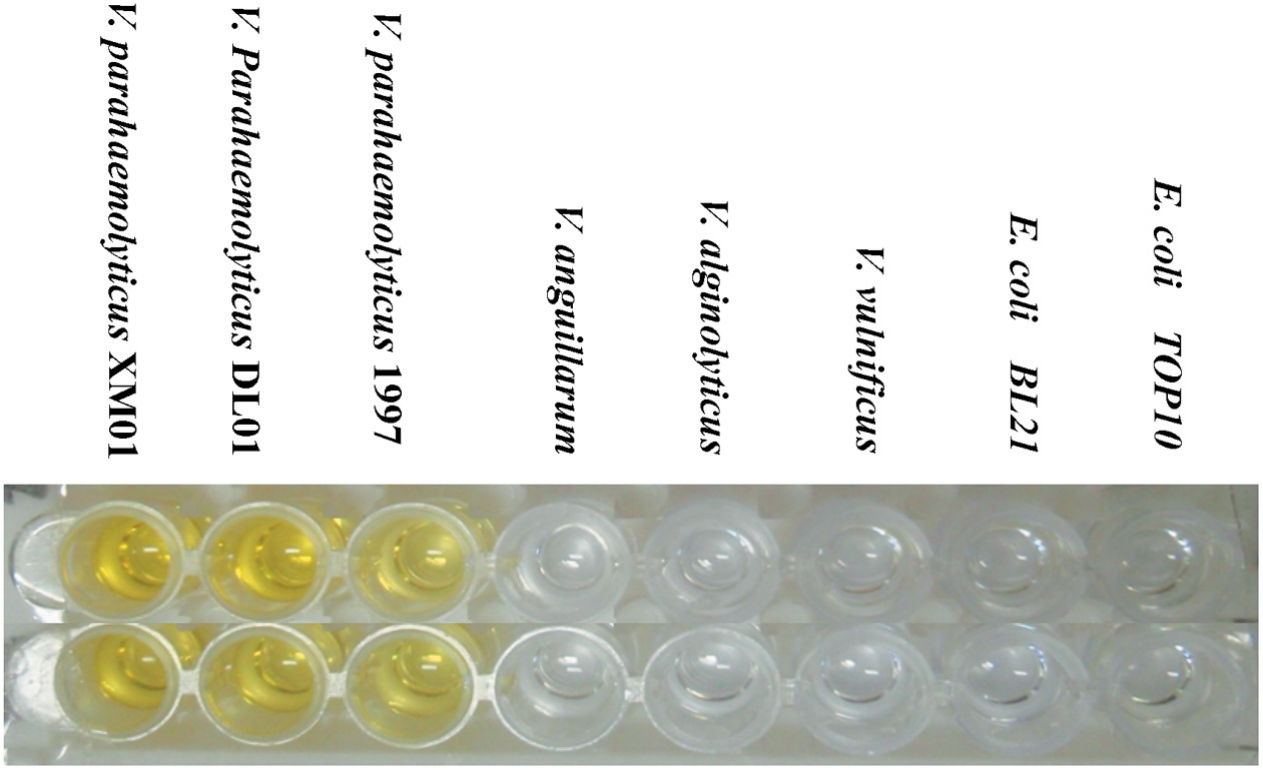
**ELISA Detection of TLH in Bacteria.** Eight different bacterial strains (three TLH positive *V. parahaemolyticus* strains, three TLH negative *Vibrio* strains and two other *E. coli* strains) were cultured in APW medium without shaking for 16 h, and the supernatant of culture was used to coat the 96 wells plate at 4°C for overnight. The ELISA assay was determined using an anti 6×His tag antibody. (PC, positive control; NC, negative control).

We were interested in determining whether the co-expressed scFv could serve as a good antibody reagent to directly detect TLH from bacterial strains. In the first detection, three TLH positive *V. parahaemolyticus* strains, three TLH negative Vibrio strains and two other *E. coli* strains were used to identify the practicality and feasibility of co-expressed scFv. As shown in Figure 6, three *V. parahaemolyticus* strains gave the positive signal, and other five non *V. parahaemolyticus* strains all gave the negative results. To further detect the accuracy of co-expressed scFv, total 22 different bacterial strains (eleven different types *V. parahaemolyticus* strains, seven Vibrio strains from other species containing no TLH, and four other bacterial strains) were tested. Table 2 showed that all the eleven *V. parahaemolyticus* strains were successfully detected by Skp co-expressed scFv. In contrast, seven Vibrio strains from other species and four other bacterial strains of non-Vibrio all gave negative detection results. These results showed that this Skp co-expressed scFv could specifically recognize TLH positive *V. parahaemolyticus* strains, and might be used as a potential reagent for *V. parahaemolyticus* diagnosis.

**Table 2:** Detection of bacterial samples^*^.

**Strains**	**TLH**	**No. of**	**Detection**
	**Content**	**tested**	**result**
			**TLH**
*Vibrio parahaemolyticus* CGMCC1.2164	+	1	+
*Vibrio parahaemolyticus* F2002	+	1	+
*Vibrio parahaemolyticus* DL01	+	1	+
*Vibrio parahaemolyticus* CGMCC1.1997	+	1	+
*Vibrio parahaemolyticus* CGMCC1.1997A	+	1	+
*Vibrio parahaemolyticus* 121	+	1	+
*Vibrio parahaemolyticus* 124	+	1	+
*Vibrio parahaemolyticus* CGMCC1.1614	+	1	+
*Vibrio parahaemolyticus*ATCC17802	+	1	+
*Vibrio parahaemolyticus* CGMCC1.1615	+	1	+
*Vibrio parahaemolyticus* CGMCC1.1616	+	1	+
*Vibrio alginolyticus* CGMCC1.1833	−	2	−
*Vibrio vulnificus* CGMCC1.1597	−	2	−
*Vibrio anguillarium* CCTCCM204066	−	2	−
*Vibrio harveyi* CGMCC1.1601	−	1	−
*Escherichia coli* BL21	−	1	−
*Escherichia coli* DH5a	−	1	−
*Escherichia coli* TOP10	−	1	−
*Escherichia coli* JM109	−	1	−

* Each strain sample had two replicates.

## Discussion

Although scFv have been used as therapeutic agents and plays a useful role in diagnosis of diseases (Saerens et al., 2008; Tong et al., 2010), but the extensive application of scFv has been limited by the misfolding of scFv, leading to a loss of binding activity and the formation of inclusion bodies. To effectively solve the problem, a variety of strategies have been employed to improve the soluble expression in different host systems. However, to date, the bottleneck of scFv soluble expression has not been resolved, and it is necessary to develop an effective method for scFv soluble expression (Esposito and Chatterjee, 2006; Kudou et al., 2011).

Co-expression of scFv with a molecular chaperon effectively improved the correct folding, and enhanced the solubility of scFv (Hayhurst and Harris, 1999; Sonoda et al., 2011). It is well known that Skp is a periplasmic chaperone that can effective improve the folding and assembly of outer membrane proteins in *E. coli* (Hayhurst et al., 2003; Sonoda et al., 2011). In this study, a double expression vector pACYC-Duet-*skp* was chosen for construction of co-expression vector pACYC-Duet-*scFv-skp*. Two target proteins were co-expressed simultaneously in a host strain, but the expression of the two target proteins were independent, and had different fusion protein tags. Compared to other expression formats, this method neither requires removal of the fusion protein tags, nor the need for co-transformation of plasmids, and makes the entire process of protein purification simple, convenient, and cost-effective. Besides, the expressed target scFv folded correctly and retained the full binding activity with the help of molecular chaperone, and the purified protein could be used directly for different purposes without refolding of protein *in vitro* after purification.

In this study, four different format expression vectors were constructed for soluble expression of scFv. Amongst the four purified protein, the Skp co-expressed scFv showed a higher solubility, and a 3–4 fold higher binding to antigen TLH than the other three purified proteins. Besides, the Skp co-expressed scFv was specific for TLH and did not cross-react with other TLH-associated proteins. Although TRX fusion expressed scFv had higher solubility, but the binding activity of TRX fusion protein was lower than Skp co-expressed scFv (Marblestone et al., 2006; Subedi et al., 2012). Together, the results demonstrate that the pACYC-Duet-*Skp* vector system is an effective expression system for soluble expression of scFv.

Low temperature is an important factor in soluble protein expression (Qing et al., 2004; Sørensen and Mortensen, 2011). To effectively express the soluble scFv, different temperatures (such as 37, 30, 25, and 16°C) were used. The result demonstrated that the soluble expression of scFv was improved greatly at low temperature (16°C), the expressed scFv had the highest solubility and binding activity (**data not shown**). This improvement of solubility can be attributed to the following three advantages of lower expression temperature: (1) The expression rate of protein is greatly reduced, leading to enhanced the folding efficiency of protein; (2) The endogenous proteases are usually inactive or have low-activity, leading to an enhancement of target protein stability (Subedi et al., 2012); (3) The metabolic pressure of host cells is decreased owing to the reduced the cytotoxicity of the expressed protein. However, the true mechanism of efficient expression at low temperature is still unclear, and additional works would be needed to understand this.

## Conclusions

We constructed four different expression vectors, and tested their ability to express the soluble scFv protein. The solubility and binding activity of the purified protein were analyzed by SDS-PAGE and ELISA, respectively. Amongst the four purified protein, the Skp co-expressed scFv showed the highest solubility, and the binding activity to antigen TLH was 3–4 fold higher than other three purified scFv. Also, the scFv obtained by Skp co-expression was specific for TLH and did not cross-react with other TLH-associated proteins. Notably, this scFv could be used to detect TLH directly in real samples, suggesting that the pACYC-Duet-*skp* vector system is an effective system for soluble expression of scFv.

## Author contributions

Rongzhi Wang, Youjun Feng, and Shihua Wang designed the experiments and wrote the manuscript. Rongzhi Wang, Shuangshuang Xiang, Yonghui Zhang performed all the experiments. DL Wu, Youjun Feng, Swaminath Srinivas, and Mingshen Lin performed a few experiments and data analysis. All authors read and approved the final manuscript.

### Conflict of interest statement

The authors declare that the research was conducted in the absence of any commercial or financial relationships that could be construed as a potential conflict of interest.

## Abbreviations

*V.p*: *Vibrio parahaemolyticus*
TLH: Thermolabile Hemolysin
IPTG: Isopropyl β-D-1-thiogalactopyranoside
scFv: single chain variable fragment.
